# Supplementary material for: Development of a Cytotoxic Antibody–Drug Conjugate Targeting Membrane Immunoglobulin E-Positive Cells
Source: Int J Mol Sci. 2023 Oct 8;24(19):14997. doi: 10.3390/ijms241914997 (PMC10573690; doi:10.3390/ijms241914997)
Supplement: Supplementary file 1 [file ijms-24-14997-s001.zip › Supplementary Figure S1.pdf]

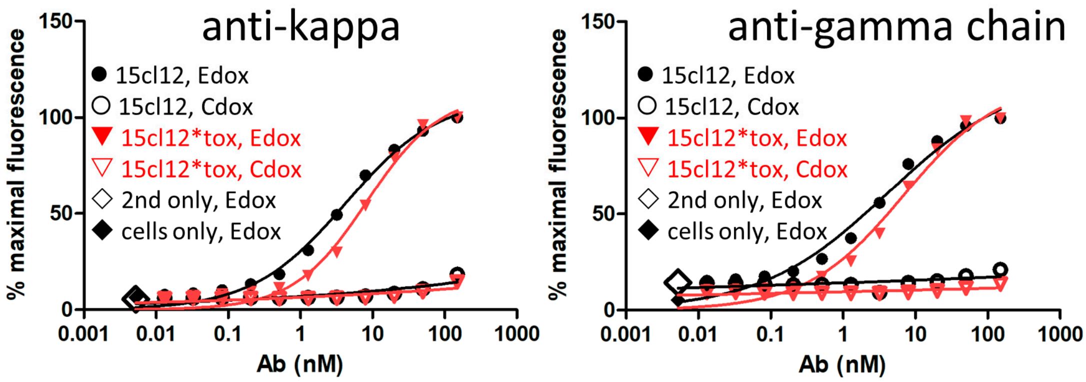

Supplementary Figure S1. Results of FACS experiment with 15cl12 and its toxin derivate binding to antigen-positive (Edox) cells and antigen-negative (Cdox) cells. The antibodies were detected with an anti-kappa conjugate (left) or an anti-Fc conjugate (right) and the percent maximal fluorescence was evaluated as: mean fluorescence intensity (MFI) of the sample/maximal MFI*100. 15cl12*tox: 15cl12_L328 coupled with toxin; 2nd only: secondary reagent only.
