# Supplementary material for: Development of a Cytotoxic Antibody–Drug Conjugate Targeting Membrane Immunoglobulin E-Positive Cells
Source: Int J Mol Sci. 2023 Oct 8;24(19):14997. doi: 10.3390/ijms241914997 (PMC10573690; doi:10.3390/ijms241914997)
Supplement: Supplementary file 1 [file ijms-24-14997-s001.zip › Supplementary Figure S2.pdf]

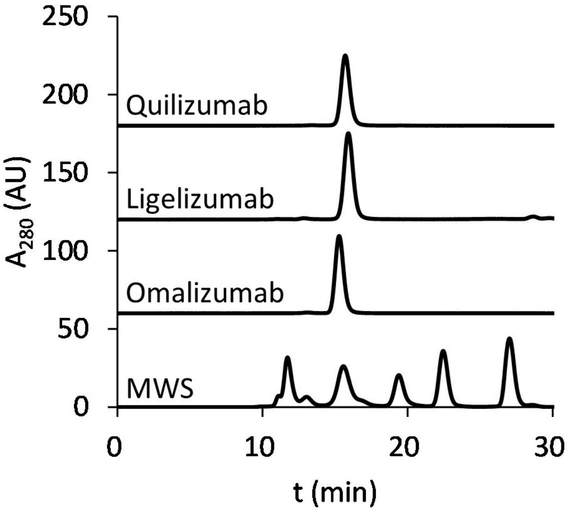


**Supplementary Figure S2.** Analytical size-exclusion chromatography in native conditions of anti-IgE antibodies.
