# Supplementary material for: Development of a Cytotoxic Antibody–Drug Conjugate Targeting Membrane Immunoglobulin E-Positive Cells
Source: Int J Mol Sci. 2023 Oct 8;24(19):14997. doi: 10.3390/ijms241914997 (PMC10573690; doi:10.3390/ijms241914997)
Supplement: Supplementary file 1 [file ijms-24-14997-s001.zip › Supplementary Figure S3.pdf]

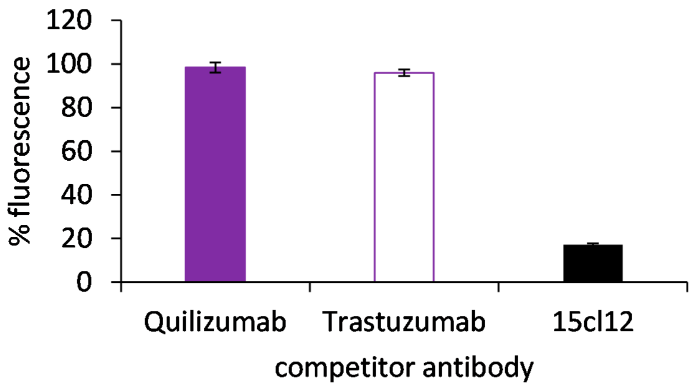

Supplementary Figure S3. 15cl12 and quilizumab do not compete for the same epitope. Edox cells were incubated with quilizumab, 15cl12 and trastuzumab as a non-binding control antibody or blocking buffer only (non-treated cells) and subsequently stained with 15cl12 labelled with Alexa Fluor 488. Percent fluorescence was calculated as: (Mean fluorescence intensity (MFI) of non-treated cells-MFI of treated cells)/MFI of non-treated cells*100.
