# Supplementary material for: Development of a Cytotoxic Antibody–Drug Conjugate Targeting Membrane Immunoglobulin E-Positive Cells
Source: Int J Mol Sci. 2023 Oct 8;24(19):14997. doi: 10.3390/ijms241914997 (PMC10573690; doi:10.3390/ijms241914997)
Supplement: Supplementary file 1 [file ijms-24-14997-s001.zip › Supplementary Figure S4.pdf]

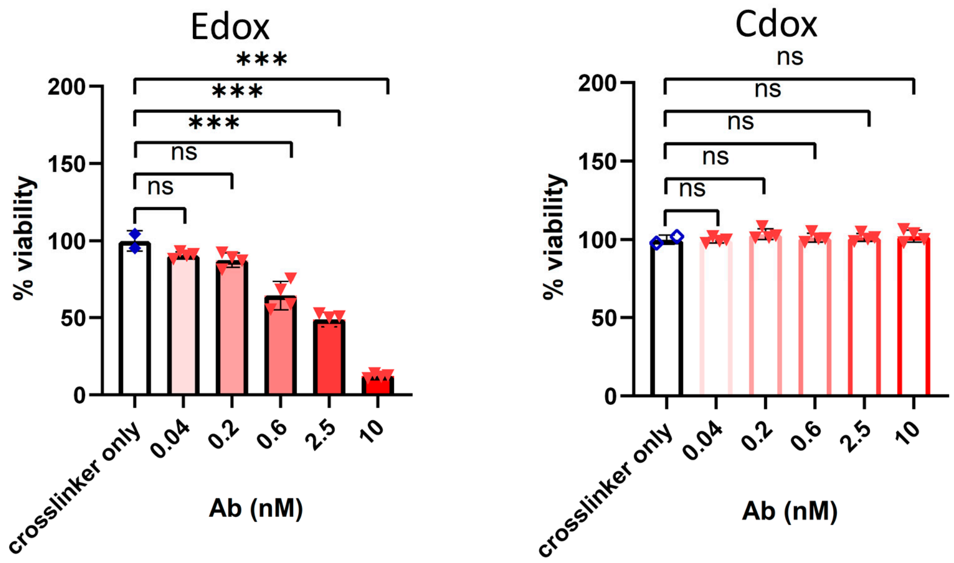

Supplementary Figure S4. Significance of the cytotoxic effect of conjugated 15cl12 antibody with added crosslinker. Data was analyzed with one-way ANOVA in Prism 8.0.2 (ns: not significant, P>0.05, ***: P<0.001).
