# Supplementary material for: Development of a Cytotoxic Antibody–Drug Conjugate Targeting Membrane Immunoglobulin E-Positive Cells
Source: Int J Mol Sci. 2023 Oct 8;24(19):14997. doi: 10.3390/ijms241914997 (PMC10573690; doi:10.3390/ijms241914997)
Supplement: Supplementary file 1 [file ijms-24-14997-s001.zip › Supplementary Figure S5.pdf]

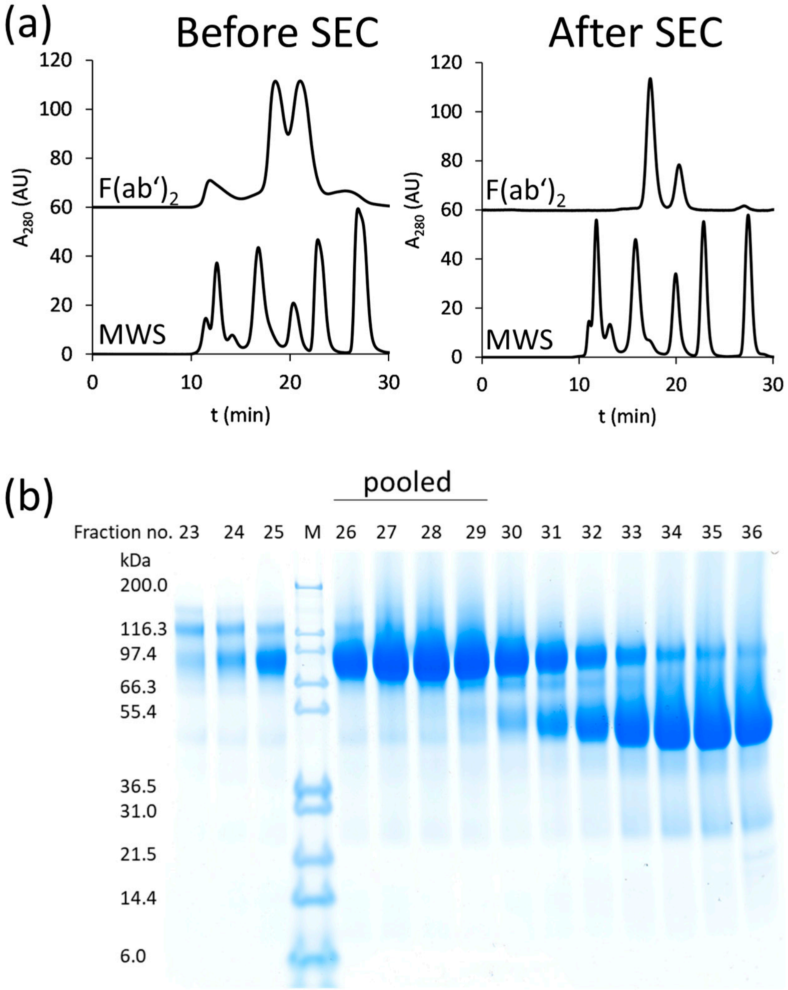

Supplementary Figure S5. Preparative gel filtration of F(ab')2 fragment used as a cross-linker for 15cl12 in cytotoxicity assays. **(a)** Size exclusion chromatography (SEC) in native conditions showing the F(ab')2 before and after preparative gel purification. MWS: molecular weight standard with proteins of 670, 158, 44, 17 and 1.3 kDa in size; **(b)** SDS-PAGE with fractions eluted after gel filtration, fractions 26-29 were pooled. M, Mark 12 Unstained Standard (Fisher Scientific).
