# Supplementary material for: Development of a Cytotoxic Antibody–Drug Conjugate Targeting Membrane Immunoglobulin E-Positive Cells
Source: Int J Mol Sci. 2023 Oct 8;24(19):14997. doi: 10.3390/ijms241914997 (PMC10573690; doi:10.3390/ijms241914997)
Supplement: Supplementary file 1 [file ijms-24-14997-s001.zip › Supplementary Table S1.pdf]

Supplementary Table S1. Amino acid sequences of all constructs used in this study.

**15cl12**
Heavy chain:<br>QIQLQQSGAELVKPGASVKLSCKASGYTFTSYDINWVRQRPERGLDWIGWIFPGDGSSKYNENFKGKATLTTDKSSNTAYIHLSR<br>LTSEDSAVYFCARWGKTFDYWGQGTTLTVSSASTKGPSVFPLAPSSKSTSGGTAALGCLVKDYFPEPVTVSWNSGALTSGVHTFP<br>AVLQSSGLYSLSSVVTVPSSSLGTQTYICNVNHKPSNTKVDKKVEPKSCDKTHTCPPCPAPEAAGGPSVFLFPPKPKDTLMISRTPE<br>VTCVVVDVSHEDPEVKFNWYVDGVEVHNAKTKPREEQYNSTYRVVSVLTVLHQDWLNGKEYKCKVSNKALPAPIEKTISKAKGQ<br>PREPQVYTLPPSRDELTKNQVSLTCLVKGFYPSDIAVEWESNGQPENNYKTTPPVLDSDGSFFLYSKLTVDKSRWQQGNVFSCSV<br>MHEALHNHYTQKSLSLSPGK
Light chain:<br>DIVMSQSPSSLAVSVGEKVTMSCKSSQSLLYSRDQKNYLAWFQQKPGQSPKLLIYWASARESGVPDRFTGSGSGTDFTLTITSLKT<br>EDLAVYYCQQFYNYPRTFGGGTKLEIKRTVAAPSVFIFPPSDEQLKSGTASVVCLLNNFYPREAKVQWKVDNALQSGNSQESVTE<br>QDSKDSTYSLSSTLTLSKADYEKHKVYACEVTHQGLSSPVTKSFNRGEC
**Omalizumab**
Heavy chain:<br>EVQLVESGGGLVQPGGSLRLSCAVSGYSITSGYSWNWIRQAPGKCLEWVASITYDGSTNYNPSVKGRITISRDDSKNTFYLQMNS<br>LRAEDTAVYYCARGSHYFGHWHFAVWGQGTLVTVSSASTKGPSVFPLAPSSKSTSGGTAALGCLVKDYFPEPVTVSWNSGALTS<br>GVHTFPAVLQSSGLYSLSSVVTVPSSSLGTQTYICNVNHKPSNTKVDKKVEPKSCDKTHTCPPCPAPELLGGPSVFLFPPKPKDTLM<br>ISRTPEVTCVVVDVSHEDPEVKFNWYVDGVEVHNAKTKPREEQYNSTYRVVSVLTVLHQDWLNGKEYKCKVSNKALPAPIEKTIS<br>KAKGQPREPQVYTLPPSRDELTKNQVSLTCLVKGFYPSDIAVEWESNGQPENNYKTTPPVLDSDGSFFLYSKLTVDKSRWQQGN<br>VFSCSVMHEALHNHYTQKSLSLSPGK
Light chain:<br>DIQLTQSPSSLSASVGDRVTITCRASQSVDYDGDSYMNWYQQKPGKAPKLLIYAASYLESGVPSRFSGSGSGTDFTLTISSLQPEDF<br>ATYYCQQSHEDPYTFGCGTKVEIKRTVAAPSVFIFPPSDEQLKSGTASVVCLLNNFYPREAKVQWKVDNALQSGNSQESVTEQDS<br>KDSTYSLSSTLTLSKADYEKHKVYACEVTHQGLSSPVTKSFNRGEC
**Ligelizumab**
Heavy chain:<br>QVQLVQSGAEVMKPGSSVKVSCKASGYTFSWYWLEWVRQAPGHGLEWMGEIDPGTFTTNYNEKFKARVTFTADTSTSTAYM<br>ELSSLRSEDTAVYYCARFSHFSGSNYDYFDYWGQGTLVTVSSASTKGPSVFPLAPSSKSTSGGTAALGCLVKDYFPEPVTVSWNSG<br>ALTSGVHTFPAVLQSSGLYSLSSVVTVPSSSLGTQTYICNVNHKPSNTKVDKKVEPKSCDKTHTCPPCPAPELLGGPSVFLFPPKPK<br>DTLMISRTPEVTCVVVDVSHEDPEVKFNWYVDGVEVHNAKTKPREEQYNSTYRVVSVLTVLHQDWLNGKEYKCKVSNKALPAPI<br>EKTISKAKGQPREPQVYTLPPSRDELTKNQVSLTCLVKGFYPSDIAVEWESNGQPENNYKTTPPVLDSDGSFFLYSKLTVDKSRWQ<br>QGNVFSCSVMHEALHNHYTQKSLSLSPGK
Light chain:<br>EIVMTQSPATLSVSPGERATLSCRASQSIGTNIHWYQQKPGQAPRLLIYYASESISGIPARFSGSGSGTEFTLTISSLQSEDFAVYYC<br>QQSWSWPTTFGGGTKVEIKRTVAAPSVFIFPPSDEQLKSGTASVVCLLNNFYPREAKVQWKVDNALQSGNSQESVTEQDSKDS<br>TYSLSSTLTLSKADYEKHKVYACEVTHQGLSSPVTKSFNRGEC
**Quilizumab**
Heavy chain:<br>EVQLVESGGGLVQPGGSLRLSCAASGFTFSDYGIAWVRQAPGKGLEWVAFISDLAYTIYYADTVTGRFTISRDNSKNTLYLQMNS<br>LRAEDTAVYYCARDNWDAMDYWGQGTLVTVSSASTKGPSVFPLAPSSKSTSGGTAALGCLVKDYFPEPVTVSWNSGALTSGVH<br>TFPAVLQSSGLYSLSSVVTVPSSSLGTQTYICNVNHKPSNTKVDKKVEPKSCDKTHTCPPCPAPELLGGPSVFLFPPKPKDTLMISRT<br>PEVTCVVVDVSHEDPEVKFNWYVDGVEVHNAKTKPREEQYNSTYRVVSVLTVLHQDWLNGKEYKCKVSNKALPAPIEKTISKAK<br>GQPREPQVYTLPPSRDELTKNQVSLTCLVKGFYPSDIAVEWESNGQPENNYKTTPPVLDSDGSFFLYSKLTVDKSRWQQGNVFS<br>CSVMHEALHNHYTQKSLSLSPGK
Light chain:<br>DIQMTQSPSSLSASVGDRVTITCRSSQSLVHNNANTYLHWYQQKPGKAPKLLIYKVSNRFSGVPSRFSGSGSGTDFTLTISSLQPE<br>DFATYYCSQNTLVPWTFGQGTKVEIKRTVAAPSVFIFPPSDEQLKSGTASVVCLLNNFYPREAKVQWKVDNALQSGNSQESVTE<br>QDSKDSTYSLSSTLTLSKADYEKHKVYACEVTHQGLSSPVTKSFNRGEC
**Cetuximab**
Heavy chain:

QVQLKQSGPGLVQPSQSLSITCTVSGFSLTNYGVHWVRQSPGKGLEWLGVIWSGGNTDYNTPFTSRLSINKDNSKSQVFFKMN<br>SLQSNDTAIYYCARALTYYDYEFAYWGQGTLVTVSAASTKGPSVFPLAPSSKSTSGGTAALGCLVKDYFPEPVTVSWNSGALTSG<br>VHTFPAVLQSSGLYSLSSVVTVPSSSLGTQTYICNVNHKPSNTKVDKKVEPKSCDKTHTCPPCPAPELLGGPSVFLFPPKPKDTLMI<br>SRTPEVTCVVVDVSHEDPEVKFNWYVDGVEVHNAKTKPREEQYNSTYRVVSVLTVLHQDWLNGKEYKCKVSNKALPAPIEKTIS<br>KAKGQPREPQVYTLPPSRDELTKNQVSLTCLVKGFYPSDIAVEWESNGQPENNYKTTPPVLDSDGSFFLYSKLTVDKSRWQQGN<br>VFSCSVMHEALHNHYTQKSLSLSPGK
Light chain:<br>DILLTQSPVILSVSPGERVSFSCRASQSIGTNIHWYQQRTNGSPRLLIKYASESISGIPSRFSGSGSGTDFTLSINSVESEDIADYYCQQ<br>NNNWPTTFGAGTKLELKRTVAAPSVFIFPPSDEQLKSGTASVVCLLNNFYPREAKVQWKVDNALQSGNSQESVTEQDSKDSTYS<br>LSSTLTLSKADYEKHKVYACEVTHQGLSSPVTKSFNRGEC
**Trastuzumab**
Heavy chain:<br>EVQLVESGGGLVQPGGSLRLSCAASGFNIKDTYIHWVRQAPGKGLEWVARIYPTNGYTRYADSVKGRFTISADTSKNTAYLQMN<br>SLRAEDTAVYYCSRWGGDGFYAMDYWGQGTLVTVSSASTKGPSVFPLAPSSKSTSGGTAALGCLVKDYFPEPVTVSWNSGALT<br>SGVHTFPAVLQSSGLYSLSSVVTVPSSSLGTQTYICNVNHKPSNTKVDKKVEPKSCDKTHTCPPCPAPELLGGPSVFLFPPKPKDTL<br>MISRTPEVTCVVVDVSHEDPEVKFNWYVDGVEVHNAKTKPREEQYNSTYRVVSVLTVLHQDWLNGKEYKCKVSNKALPAPIEKT<br>ISKAKGQPREPQVYTLPPSRDELTKNQVSLTCLVKGFYPSDIAVEWESNGQPENNYKTTPPVLDSDGSFFLYSKLTVDKSRWQQG<br>NVFSCSVMHEALHNHYTQKSLSLSPGK
Light chain:<br>DIQMTQSPSSLSASVGDRVTITCRASQDVNTAVAWYQQKPGKAPKLLIYSASFLYSGVPSRFSGSRSGTDFTLTISSLQPEDFATYY<br>CQQHYTTPPTFGQGTKVEIKRTVAAPSVFIFPPSDEQLKSGTASVVCLLNNFYPREAKVQWKVDNALQSGNSQESVTEQDSKDS<br>TYSLSSTLTLSKADYEKHKVYACEVTHQGLSSPVTKSFNRGEC
